# Water-Use Efficiency: Advances and Challenges in a Changing Climate

**DOI:** 10.3389/fpls.2019.00103

**Published:** 2019-02-19

**Authors:** Jerry L. Hatfield, Christian Dold

**Affiliations:** National Laboratory for Agriculture and the Environment, Agricultural Research Service, United States Department of Agriculture, Ames, IA, United States

**Keywords:** transpiration, energy balance, carbon dioxide, photosynthesis, agronomic practices, temperature, carbon dioxide–analysis, biomass

## Abstract

Water use efficiency (WUE) is defined as the amount of carbon assimilated as biomass or grain produced per unit of water used by the crop. One of the primary questions being asked is how plants will respond to a changing climate with changes in temperature, precipitation, and carbon dioxide (CO_2_) that affect their WUE At the leaf level, increasing CO_2_ increases WUE until the leaf is exposed to temperatures exceeded the optimum for growth (i.e., heat stress) and then WUE begins to decline. Leaves subjected to water deficits (i.e., drought stress) show varying responses in WUE. The response of WUE at the leaf level is directly related to the physiological processes controlling the gradients of CO_2_ and H_2_O, e.g., leaf:air vapor pressure deficits, between the leaf and air surrounding the leaf. There a variety of methods available to screen genetic material for enhanced WUE under scenarios of climate change. When we extend from the leaf to the canopy, then the dynamics of crop water use and biomass accumulation have to consider soil water evaporation rate, transpiration from the leaves, and the growth pattern of the crop. Enhancing WUE at the canopy level can be achieved by adopting practices that reduce the soil water evaporation component and divert more water into transpiration which can be through crop residue management, mulching, row spacing, and irrigation. Climate change will affect plant growth, but we have opportunities to enhance WUE through crop selection and cultural practices to offset the impact of a changing climate.

## Introduction

**FIGURE 1:**
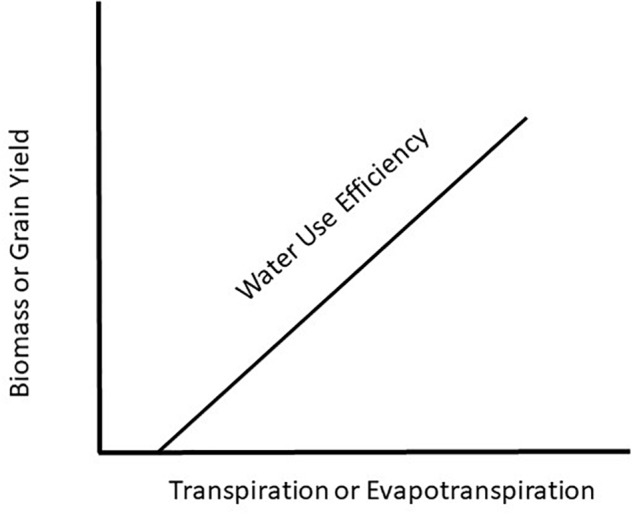
Generalized view of water use efficiency as a function of the water use by a crop relative to biomass or grain production.

Water use efficiency (WUE) is a concept introduced 100 years ago by Briggs and Shantz (1913) showing a relationship between plant productivity and water use. They introduced the term, WUE, as a measure of the amount of biomass produced per unit of water used by a plant. Since that time, there have been countless original papers and reviews written on the topic with the most recent one by Basso and Ritchie (2018) demonstrating that maize (*Zea mays* L.) productivity could be increased with no change in water use rate and result in increased WUE. This is a critical observation because the prevailing hypothesis for WUE (Figure 1) is based on plant productivity increasing with increasing water use and in order to increase WUE will require increased crop water use. To understand how WUE could be affected by a changing climate it will be necessary to determine how climate change will impact plant growth and water use of the plant. To achieve this understanding requires we examine WUE at the leaf, plant, and canopy level in response to a changing climate.

Throughout this review we will address the potential changes in WUE at multiple levels of plants to determine where potential improvements could be made in WUE. If we examine concept of water use by the plant there is a difference among the processes that occur at the leaf level compared to the canopy level. At the leaf level, water use is controlled by the available energy impinging on the leaf, vapor pressure deficit, and aerodynamic exchange, but, regulated by stomatal conductance (*g*_s_). While at the canopy level, the processes involve energy exchange at the soil surface and the plant canopy and water loss is a combination of evaporation from the soil surface and transpiration from the plant canopy. The combination of evaporation and transpiration is referred to as evapotranspiration (ET) and in the literature on WUE of plants, there is extensive use of crop water use as the metric for WUE. These specific terms need to be carefully evaluated when interpreting the results obtained from different studies or comparing among studies.

## Changing Climate

There are four factors changing in the climate that will affect water use by plants. These factors are: increasing carbon dioxide (CO_2_) concentrations, increasing temperatures, more variable precipitation, and variations in humidity. Projections of climate change are a result of a combined set of simulation models using various scenarios of changes in carbon dioxide (CO_2_) concentrations and the associated forcing functions (Collins et al., 2013). The current CO_2_ concentrations are nearly 400 ppm in 2018 and projected to increase to a range of 794–1142 ppm by 2100 without any abatement scenarios (Collins et al., 2013). These findings have been summarized based from the reports by Trenberth (2011) and Collins et al. (2013) as:

(1) Global mean temperatures will increase throughout the 21st century if CO_2_ concentrations continue to increase and under the highest CO_2_ emission scenario increases range from 2.6 to 4.8°C.
(2) Temperatures changes will not be uniform across regions with land surfaces warming more than over the oceans.
(3) Increasing global temperatures will result in more hot extremes and fewer cold extremes at both daily and seasonal time scales.
(4) Precipitation will increase with increases in global mean surface temperature and could increase 1–3% °C^-1^; however, there will be substantial spatial variation in these changes.
(5) The water holding capacity of air increases by 7% °C^-1^. The air can take up more water, and water vapor inclines. That leads to higher intensity of precipitation, i.e., higher amount of rainfall per rain event.
(6) Annual surface evaporation will increase with temperatures increases; however, over land, evaporation will be linked to precipitation.

These changes in climate will increase atmospheric water demand by crops and increase the potential for limitations in soil water availability, because of the increased variation in precipitation during the growing season and even more so in soils with limited water holding capacity. For example, Xiao et al. (2013) observed that the spatial patterns in carbon and water fluxes were dependent upon annual temperatures, precipitation, and growing season length when they compared these fluxes across a range of latitudes using eddy covariance flux systems. These types of comparisons identify the factors related to WUE of different ecosystems, and they found WUE was related to annual precipitation, gross primary productivity (GPP), and growing season length (Xiao et al., 2013). In their comparison, forests and coastal wetlands had a higher WUE than grasslands and croplands. Guoju et al. (2013a) found that in China, WUE of maize (*Z. mays* L.), wheat (*Triticum aestivum* L.), and potato (*Solanum tuberosum* L.) increased over the past 50 years that they attributed to an increase in temperature and a decrease in precipitation. Projected changes in climate are expected to increase the areas subjected to drought around the world (Dai, 2013; Feng and Fu, 2013; Fu and Feng, 2014; Huang et al., 2015). The effect of increasing drought on net primary productivity has been seen by Zhao and Running (2009) where they found a reduction of 55 petagrams of carbon due to drought. Drought will impact productivity and throughout this paper, we will explore the mechanisms of avenues to increase WUE of agricultural systems to take advantage of a limited water supply.

## Leaf Level Processes

One way to explore the impact of a changing climate on WUE is to begin at the leaf level. The interactions of a changing CO_2_ and water and temperature regimes will be most evident at the leaf level because there are not the confounding effects of canopy architecture or the interactions of the soil environment on WUE. There have been two ways proposed to calculated leaf level WUE. The instantaneous WUE is calculated as the net photosynthetic rate (*A*_n_) divided by transpiration rate (*E*). Another measure is the intrinsic WUE, which is calculated as *A*_n_ divided by *g*_s_.

Leaf level WUE has a distinctive pattern depending on the carboxylation pathway, i.e., C_3_ photosynthesis, C_4_ photosynthesis, and Crassulacean acid metabolism (CAM). C_4_ plants have higher intrinsic WUE than C_3_ plants, owing to higher *A*_n_ and lower *g*_s_ (Taylor et al., 2010). A comparison of C_3_ and C_4_ plants with Crassulacean acid metabolism (CAM) reveals a completely different pattern of stomatal response to environmental conditions. Males and Griffiths (2017) provide an overview of the stomatal processes in CAM plants and the advantages in arid environments. Bartlett et al. (2014) proposed a model to describe the storage of malic acid in CAM plants and how this responded to rubisco dynamics in the leaf carbon assimilation model. The WUE of CAM plants is quite high compared to C_3_ and C_4_ plants because of this unique cycle of carbon fixation and storage during the diurnal cycle. Yang et al. (2015) proposed a series of potential studies to enhance the understanding of the potential utilization of CAM plants as part of the food security pathways under a changing climate.

### Heat and Water-Deficit Stress, and Radiation Limitations

Climatic changes may induce or ameliorate abiotic stress to the plant, that is (1) water-deficit stress and (2) heat stress. The combined effect of heat and water-deficit stress on plant productivity have been summarized by Hatfield et al. (2011) for crops and Izaurralde et al. (2011) for range and pasture plants. Hatfield et al. (2018) in evaluating the cause for yield reductions in the Midwest found a combination of high temperatures during the pollination and grain-filling period coupled with water-deficit stress induced by below normal precipitation during the grain-filling period explained yield variation among years. Water-deficit stress may be induced by changes in available water and vapor pressure deficit (VPD). Heat stress may be induced by increased ambient air temperature and in the absence of water-deficit stress will decrease productivity (Hatfield, 2016).

There have been numerous assessment of the effects of increased temperatures or drought on the productivity of crops (Long and Ort, 2010; Lobell et al., 2011, 2013, 2014). A novel observation by Vanzo et al. (2015) showed a difference in the isoprene emissions from poplar (*Populus* spp.) and found differences between emitting (IE) and non-emitting (NE) isoprene plants. When exposed to hot, dry conditions, the chloroplastidic electron transport rate of NE plants became impaired, while IE plants maintained values similar to unstressed controls. During recovery from hot, dry exposures, IE plants reached higher daily net CO_2_ assimilation rates compared with NE genotypes. Examining the changes in volatile emissions from plants coupled with observations on the enzymatic activity may begin to reveal the differences among plants in their response to high temperatures, water deficits, as well as fluctuating light regimes. Leaf level responses are complex because of the internal changes in enzymatic activity in response to the environment. Illustrative of this is the recent observations by Slattery et al. (2018) in which they observed Rubisco, Rubisco activase (Rca), glyceraldehyde-3-phosphate dehydrogenase (GAPDH), Fru-1,6-bisphosphatase (FBPase), sedoheptulose-1,7-bisphosphatase (SBPase), and phosphoribulokinase (PRK) where changed in response to changing light. These abiotic stresses are connected and additionally interact with increasing CO_2_.

### Increase of Ambient CO_2_ Concentration

**FIGURE 2:**
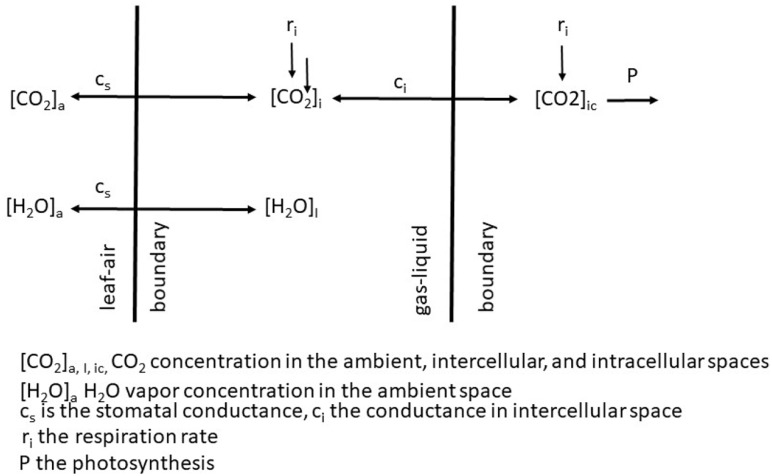
Schematic of the exchange of CO_2_ and H_2_O vapor across from the ambient air to the intercellular spaces of a leaf.

**FIGURE 3:**
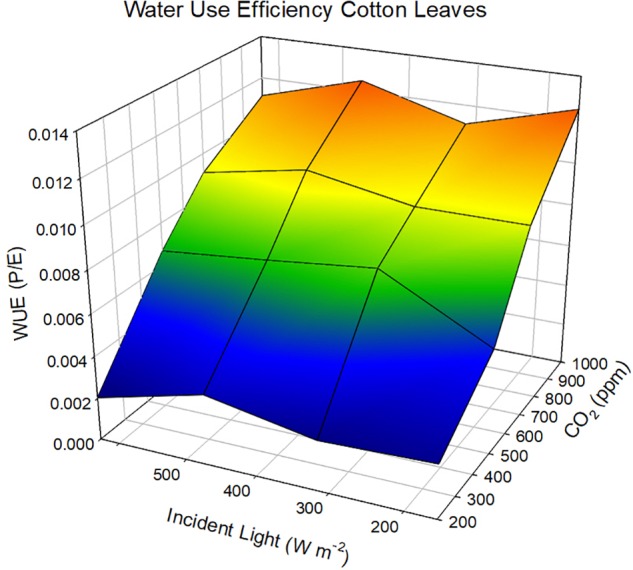
Response of water use efficiency in cotton leaves as a function of changing CO_2_ and incident light levels at a constant wind speed of 2.4 cm sec^-1^. Data redrawn from Bierhuizen and Slatyer (1965).

The sole effect of increasing CO_2_ on *A*_n_ and WUE is generally positive because the gradient between the ambient air and the intercellular spaces is increased and in the presence of light, CO_2_ within the leaf is rapidly converted to carbohydrates. If we adopt the kinetic model described by Charles-Edwards (1971) as redrawn in Figure 2 then the linkages between CO_2_ uptake and water loss by a leaf become apparent. The governing factors in this kinetic model are the diffusion coefficient, which is analogous to *g*_s_. When we compare the exchange processes of CO_2_ within the leaf and H_2_O vapor then the dynamics of the exchange processes are controlled by *g*_s_ for water vapor and *g*_s_ and mesophyll conductance (*g*_m_) for photosynthesis (Lawson and Blatt (2014). This is also why WUE increases under water-deficit stressed conditions – the reduction in *A*_n_ is less than the reduction of *E* or *g*_s_. Earl (2002) found no significant difference in *A*_n_, but lower *g*_s_ in soybean genotypes with higher WUE. Bierhuizen and Slatyer (1965) were among the first to explain the relationship observed between *E* and *A*_n_ for different species due the change in saturation deficit and CO_2_ concentration. They showed there was an increase in WUE of cotton (*Gossypium hirsutum* L.) with increasing CO_2_ levels across all light levels impinging on the leaf (Figure 3). However, there is a different response to rising CO_2_ among C_3_ photosynthesis and C_4_ photosynthesis plants. A beneficial effect is observed in C_3_ plants because CO_2_ is a limiting factor owing to the functioning of the carboxylation pathway. C_4_ plants show little effect on increased CO_2_ under optimal soil water conditions; only under drought stress high CO_2_ levels is beneficial owing to partial stomate closure thus reducing transpiration, and the ability of C_4_ plants to assimilate carbon even when stomates are closed (Lopes et al., 2011).

### Leaf Level Interactions Among Climatic Parameters and CO_2_

Yoo et al. (2009) provided an overview of the role of changing transpiration on crop water use and understanding the combined response of climate impacts on carbon assimilation and water use will be key to quantifying the effects of a changing climate. It is therefore significant to observe the interactions of CO_2_, temperature and water regime to understand WUE in a changing climate.

While increased CO_2_ can ameliorate water-deficit stress, it cannot offset the increase in heat stress, and may even be adverse, because *E* decreases and leaf temperature increases (Lopes et al., 2011). Allen et al. (2003) used soybean [*Glycine max* (L.) Merr.] to evaluate the effect of CO_2_ and temperature on WUE, foliage temperature, and canopy conductance. They used a combination of air temperatures in small chambers to expose soybean leaves to a range of temperatures, VPDs, and CO_2_ concentrations. Leaf conductance did not show any response to increasing CO_2_ but were affected by temperature with the lower conductance evident with the higher temperatures. Water use was not affected by increasing CO_2_ but was increased with the higher temperatures. The overall result was the WUE decreased with the increasing temperatures but increased with increasing CO_2_ at each temperature regime.

Another complicating factor in this type of experiment is the changing VPD of the air with changing temperature and the feedback effect on leaf temperature. The change in the air temperature surrounding the leaf will change leaf temperature and directly affect the gradient of water vapor between the leaf and the atmosphere. This gradient is affected by the internal leaf water vapor pressure (e; kPa) which is coupled to leaf temperature (T; °C) and can be calculated from Tetens’ equation (Monteith and Unsworth, 2013):

(1) e=0.61078*exp [17.269*T/(T+237.3)]

Atmospheric factors affecting the energy balance and leaf or canopy temperature drive internal water vapor pressure and ultimately water use. Increases in air temperature will directly increase crop canopy temperature, leaf water vapor pressure, and transpiration. The response shown in Figure 3 would be expected at the leaf level because the uptake of CO_2_ is controlled more by the concentration gradient from the leaf to the air than *g*_s_ or the diffusion coefficient. The CO_2_ concentration gradient is large because the internal concentration at the mesophyll is near zero creating a large gradient from the ambient air into leaf. This is in contrast to the H_2_O vapor gradient, which is at saturation just inside of the stomatal guard cell and a water vapor concentration dictated by air temperature and specific humidity. The differences in these two gradients reveal that leaves would be more efficient in the photosynthetic process than the transpiration process and would exhibit a preferential shift toward greater WUE at the leaf level because *A*_n_ would be affected more than *E*. El-Sharkawy and Cock (1984) compared different cassava (*Manihot esculenta* Crantz) cultivars and found WUE decreased with increasing VPD. If we extend this across species and climate change scenarios, then humidity of air in response to changing temperatures will have a significant impact on WUE. One has to be cautious of the older literature because the effect of a rapidly changing CO_2_ was not part of the research assessments and water availability, temperature, and humidity were the main variables.

### Genetic Response to WUE

The concept of WUE, alongside with other parameters, had been proposed in plant breeding to identify water use efficient genotypes under changing climate regimes, heat and water-deficit stress, and interactions among them. Variation among genotypes for WUE has been found in a number of crop species, including barley (Hubick and Farquhar, 1989), cowpea [*Vigna unguiculata* (L.) Walp.] (Ismail and Hall, 1992; Ashok et al., 1999), peanut (*Arachis hypogaea* L.) (Hubick et al., 1988; Wright et al., 1994), sorghum [*Sorghum bicolor* (L.) Moench.] (Donatelli et al., 1992), soybean (Mian et al., 1996; Hufstetler et al., 2007), upland cotton and pima cotton (*G. barbadense* L.) (Quisenberry and McMichael, 1991; Saranga et al., 2004; Fish and Earl, 2009), and wheat (Ehdaie and Waines, 1993; Van Den Boogaard et al., 1997; Siahpoosh et al., 2011; Siahpoosh and Dehghanian, 2012). In a recent meta-analysis, Gago et al. (2014) found that WUE at the leaf level is a complex trait dependent upon physiological responses that link *g*_m_ and *g*_s_ with the key variable being the factors that affect the photosynthetic process at the leaf scale. Flexas et al. (2014) had proposed the ratio of *g*_m_/*g*_s_ as the key variables related to uptake of CO_2_ by the leaf. Gago et al. (2014) found that respiration rates were a key factor in WUE because increasing respiration decreased the net uptake of carbon (C) by the leaf. They proposed that genetic screening of plants for characteristics directly related to photosynthetic efficiency or reduced respiration would lead to insights in the potential impacts of climate change on WUE. Peng and Krieg (1992) in comparing genotypes of grain sorghum found that differences in WUE among genotypes was related to *A*_n_ and leaf area because there was little difference among genotypes in their water use. In peanut (*A. hypogaea* L.), Craufurd et al. (1999) found WUE was affected by specific leaf area (leaf thickness) and carbon isotope discrimination with differences between Virginia peanut (*A. hypogaea* L. spp. fastigiata) and Spanish peanut (*A. hypogaea* L. spp. hypogaea) genotypes. In these experiments, there was an interaction between WUE and high temperatures because of the effect on specific leaf area and proposed that specific leaf area could be a parameter useful for screening among genotypes for WUE. While Ismail and Hall (1992) proposed that carbon isotope discrimination was a good selection criteria in cowpea [*V. unguiculata* (L.) Walp.]. In their study comparing these genotypes, they found a 19% variation under wet conditions and a 23% variation under dry conditions. Ramirez Builes et al. (2011) compared six genotypes of common bean under water-deficit stress conditions in the greenhouse and found differences in transpiration efficiency and WUE and were able to extend these results to show differences in WUE when beans were grown in the field environment. Similar results were found by Siahpoosh et al. (2011) for bread wheats with WUE ranging from 5.092 and 7.296 kg ha^-1^ mm^-1^ depending upon the level of water-deficit stress imposed on the cultivars. They proposed that total biomass produced and cultivar ET during the season was a valuable screening tool (Siahpoosh et al., 2011). Hufstetler et al. (2007) and Fish and Earl (2009) used epidermal conductance of dark adapted soybean and cotton leaves as a phenotypic trait related to WUE and found a negative relationship between epidermal conductance and WUE. Kromdijk et al. (2016) found under fluctuating light conditions that the interconversion of violaxanthin and zeaxanthin in the xanthophyll cycle led to increasing productivity by 15% and suggests that screening for plant response under variable light may provide insights into increasing the photosynthetic efficiency. Being able to identify traits related to WUE will aid in being able to screen across genetic material for their response under a changing climate. Comparisons among species and within species is not new, Brown and Simmons (1979) demonstrated that apparent photosynthesis and transpiration under water-deficit conditions were related to WUE and could be used as tools to assess genetic material. Gebrekirstos et al. (2011) used wood carbon isotope composition as a proxy for WUE in tree species for ∼30 years. The authors identified different drought strategies among species, which could help to draw conclusions on future climate change adaption. Song et al. (2015) could show that WUE changes with tree age using the carbon isotope method on Mongolian pine (*Pinus sylvestris* var. *mongolica*).

There has also been criticism of using leaf level WUE to identify water efficient plants. One drawback is the difficulty to upscale from leaf to canopy level (see also section below). Medrano et al. (2015) cautioned against using leaf measurements of WUE to scale to whole canopy WUE because of the potential confounding effect of leaf position relative to the photosynthetically active radiation (PAR) regime and water demand on the leaf. The microclimate surrounding an individual leaf will determine the WUE which suggests that if leaves are being used to relate to canopy level responses then a composite of leaves be used that would more closely represent the canopy. Blum (2009) dismissed the WUE-concept for plant breeding because genotypes can only increase leaf level WUE by activating plant traits responsible for reducing *E*, not by increasing *A*_n_. This would eventually lead to genotypes with reduced yield and drought tolerance. Instead, the author proposed to evaluate the Effective Use of Water (EUW) which focuses on genotypes, which are capable to maximize soil moisture capture for transpiration. There continue to be advances in our understanding of plant response to a changing CO_2_ environment, one of these responses is a change in the stomatal density as observed by Caine et al. (2019) in rice and the evolution toward changes in stomatal density from C_3_ to C_4_ plants (Way et al., 2014). The use of more advanced techniques, e.g., carbon isotope discrimination (Cernusak, 2018; Gao et al., 2018a) or molecular genetics (Avramova et al., 2018) promise to offer new insights into understanding these linkages.

## Plant and Canopy Level Responses

**FIGURE 4:**
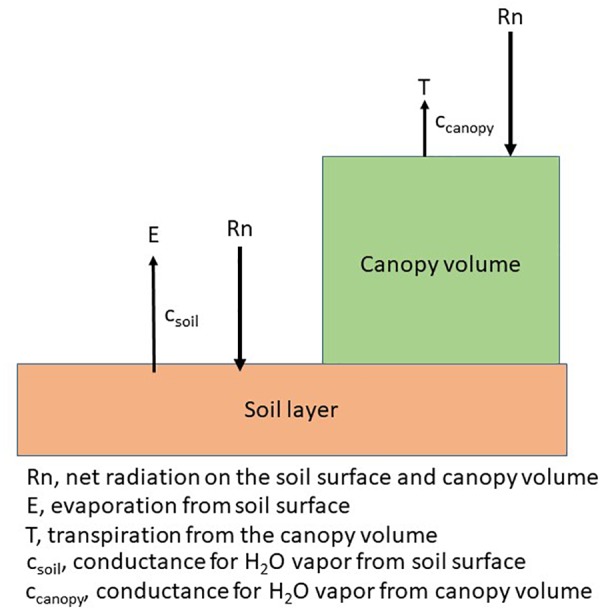
Schematic of the energy distribution on the plant and soil surface and the canopy level conductances for a canopy arranged in rows.

Each plant species has a unique arrangement of a set of single leaves and canopies consist of an arrangement of plants according to a specific cultural practice, e.g., row spacing. The arrangement of plants creates a diverse exposure to solar radiation, i.e., PAR regimes on the leaves of the plant and on the soil as shown in the following diagram (Figure 4). Plant water use, or transpiration will be governed by a combination of physiological and morphological characteristics (Kimball, 2007) while soil water evaporation is dependent upon the energy at the soil surface (Ritchie, 1972). Photosynthesis and ultimately, dry matter production, will be impacted by the interception of PAR by the canopy. In annual crops, this creates a series of unique microclimates within the canopy throughout the growing season while in perennial crops, the changes throughout the growing season may not be as large because of the more constant canopy size.

As the plant canopy develops during the growing season, the increases in leaf area are proportional to the growth rate and transpiration increases linearly with leaf area (Ritchie, 1972). As the leaf area index (LAI) of the canopy approaches 4, *E* increases at a slower rate because there is complete light interception by the canopy. Total canopy photosynthesis exhibits a similar response with a diminishing rate of canopy photosynthesis at LAI > 4. At this point in growth and development, *A*_n_ and *E* are directly related to the energy available to the canopy. Mutual shading and interference among leaves become dominant factors in determining the rate of change in photosynthesis and transpiration and there is an uncoupling of these responses from changes in LAI above 4 (Ritchie, 1972; Villalobos and Fereres, 1990; Sau et al., 2004).

### Carbon and Water Dynamics on a Canopy Level

Growth of plants and the accumulation of C into plant material on a canopy and ecosystem level is described by GPP and net ecosystem productivity (NEP). The GPP in terrestrial ecosystems is defined as the total amount of C assimilated by photosynthetic activity of plants. High GPP in the Northern Hemisphere is generated in the United States Corn Belt due to the large-scale cultivation of maize, which is a C_4_ plant (Guanter et al., 2014). Although maize is often grown in rotation with soybean to utilize the nitrogen remaining by the legume crop, Dold et al. (2017) found that maize had a positive carbon balance while soybean showed a negative balance. The net primary production (NPP) is the sum of GPP and C losses by autotrophic respiration (i.e., plant respiration; RA) (Noble et al., 2000):

(2) GPP=NPP−RA

Note the sign convention; fluxes from the atmosphere to the biosphere are positive, and *vice versa*. Hence, minimum GPP and maximum RA is zero, while NPP can be positive or negative. There have been alternative sign conventions and thresholds proposed (Roxburgh et al., 2005). The global NPP is estimated to 60 Gt C yr^-1^, meaning that about half of C assimilated is lost by autotrophic respiration (Noble et al., 2000). The net ecosystem production (NEP) is calculated as the sum of GPP and ecosystem respiration (RE) (e.g., Dold et al., 2017):

(3) GPP=NEP−RE

The RE is defined as the sum of RA and heterotrophic respiration, that is the soil mineralization from the edaphon and decomposition of dead organic material. The global NEP is estimated to 10 Gt C yr^-1^. The Net Biome Production (NBP) is the amount of C stored in a biome or an ecosystem and can be calculated as the difference between NEP and the amount of C introduced in the biome (e.g., organic fertilizer), and leaving the system (e.g., yield, dissolved carbon in water, fire). The NBP varies among biomes and is approximated globally to ±1 Gt yr^-1^ globally (Noble et al., 2000).

One of the most used methods for quantifying crop water use has been through the Penman–Monteith equation (Allen et al., 1998, 2005):

(4) $$\lambda ET=\frac{\Delta \;*\;(R_{n}-G)\;+\;p_{a}*C_{p}*\frac{VPD}{r_{a}}}{\Delta \;+\;\mathrm{\gamma *}\left(1+\frac{r_{s}}{r_{a}}\right)}$$

Where: λET = Latent heat flux (MJ m^-2^ s^-1^), *R*_n_ = net radiation (MJ m^-2^ s^-1^), G = soil heat flux (MJ m^-2^ s^-1^), *p*_a_ = mean air density at constant pressure (kg m^-3^), *C*_p_ = specific heat of air (MJ kg^-1^ °C^-1^), Δ = slope of saturation vapor pressure – temperature relationship (kPa °C^-1^), VPD = vapor pressure deficit (kPa), γ = psychrometric constant (kPa °C^-1^), *r*_s_ = surface resistance (s m^-1^), *r*_a_ = aerodynamic resistance (s m^-1^).

Total crop water use can be separated into the soil water evaporation component and the transpiration component and when combined represent ET or typically what is referred to as crop water use. As we begin to examine WUE at the canopy scale, it becomes important to understand how climate affects each of these components in the soil-plant-atmosphere continuum.

At the leaf level there is a direct relationship to WUE induced by increasing CO_2_ because of the concurrent increase in *A*_n_ and reduction in *g*_s_. Extending from the leaf to canopy level, the direct relationships between WUE and changes in climate parameters are less obvious and often not detectable (Polley, 2002). The primary reason for this lack of response is related to the temperature response of a given species and the relationship of growth response to a change in temperature and water use rate by the plant canopy (Polley, 2002). To estimate WUE at the canopy level requires a methodology to quantify the accumulation of dry matter and the water use by the canopy. One way to estimate WUE is to divide the GPP by ET, by that incorporating H_2_O and CO_2_ exchange at both, the soil surface and the plant canopy. Beer et al. (2009) tried to couple WUE to the plant canopy by multiplying canopy level WUE with daylight VPD as a proxy for canopy conductance and called it the inherent water use efficiency (IWUE^∗^). Another WUE method is the ratio of biomass accumulated or yield produced to water used (e.g., Monteith et al., 1991; Droppelmann et al., 2000; Bai et al., 2016). Bai et al. (2016) also used the Water Equivalent Ratio (WER) to estimate WUE in mixed cropping systems.

(5) $$WER=\frac{WUE_{int,A}}{WUE_{mono,A}}+\frac{WUE_{int,B}}{WUE_{mono,B}}$$

Where: WUE_mono_ is the monocropped WUE, and WUE_int_ is the intercropped WUE of crop A and B, respectively.

A WER > 1 would indicate that water use was lower in the mixed stand compared to the sole crop and *vice versa*.

### Impact of Elevated Atmospheric CO_2_ on Plant Water Use

The effect of increased CO_2_ on seasonal crop water use was observed by Bernacchi et al. (2007) when they found the control plots extracted the available soil water and the crop become water limited. In contrast, in the elevated CO_2_ plots the stomata remained open and the plants continued to transpire because of the water conservation induced by the elevated CO_2_. Soybean grown under elevated CO_2_ continued to photosynthesize and grow longer while the control plants ceased growth. Under rain-fed agriculture, which often experiences short periods of drought, the net impact of elevated concentrations of CO_2_ would enable soil water conservation, thus sustaining crop productivity for more days than under current CO_2_ concentrations.

Growth of C_3_ species with a doubling of atmospheric CO_2_ above present-day levels will increase growth nearly 30% under optimum temperature and water availability (e.g., Kimball, 1983, 2007; Kimball and Idso, 1983; Kimball et al., 2002). With a doubling of CO_2_ in soybean, *g*_s_ decreased about 40% (Ainsworth et al., 2002; Ainsworth and Rogers, 2007). Ainsworth and Long (2005) utilized free-air CO_2_ enrichment studies to evaluate the response of two C_3_ and C_4_ species to increased CO_2_ concentrations from present to 550–600 μmol mol^-1^ and found no significant differences between species with an average reduction in *g*_s_ of 20%. As the *g*_s_ decreases there is a concurrent decrease in water lost to the atmosphere. The expected changes in CO_2_ over the remainder of this century would decrease transpiration and have a positive impact on plant WUE directly related to *g*_s_ changes.

Given the fact that *T* is closely linked with crop growth and reaches a maximum after canopy closure (LAI > 4), we can expect the effects of CO_2_ on leaf area changes to be relatively small. The most significant factor is the duration of green leaf area of the plant canopy because water use will be in direct relationship to how long the leaf area persists during the growing season. However, as summarized by Hatfield et al. (2011) and Hatfield and Prueger (2015) increasing temperatures will increase the rate of development. This effect is extremely evident during the reproductive stage of crops when exposure to high temperatures hastens the rate of maturity and this is in the crop growth cycle with the maximum water use (Hatfield, 2016). The net result of increasing temperatures would be a reduction in the seasonal water use because of the shorten duration of leaf area and shortened growth cycle. A shift toward crops with a longer growing season or perennial crops would increase the seasonal crop water use because of the longer leaf area duration. Any of these crops, when exposed to increased CO_2_ exhibits decreased *g*_s_ (Kimball and Idso, 1983; Morison, 1987; Wand et al., 1999; Allen et al., 2003).

### Canopy Level Interactions of Elevated CO_2_ and ET

At the leaf scale, increasing CO_2_ results in water conservation; however, at the whole plant, canopy, or ecosystem scale these responses have diminished because of the factors that affect ET become more dominant than conductance (Field et al., 1995; Polley, 2002). The results have been variable across a range of crops on the change in ET with changing CO_2_, these results are dependent upon whether the experiments were conducted in controlled versus field environments. Hui et al. (2001) observed an increase in ET with increasing CO_2_ levels. Jones et al. (1985) observed a 12% reduction in seasonal transpiration and 51% increase in WUE when grown in ambient and doubled CO_2_. Observations in wheat (*T. aestivum* L.) by André and Du Cloux (1993) showed an 8% decrease in transpiration to doubled CO_2_, while Hunsaker et al. (1996, 2000) found a 4% reduction in ET with a 200 μmol mol^-1^ CO_2_ increase in a free air CO_2_ studies when water and N were limiting. In contrast, cotton (*G. hirsutum* L.) showed no change in ET in a similar experiment (Hunsaker et al., 1994) that they attributed to the greater growth response in cotton. However, Reddy et al. (2000) found transpiration in cotton decreased by 8% with a doubling of CO_2_. Free –air CO_2_ experiments conducted in Illinois on soybean grown at 375 and 550 μmol mol^-1^ revealed an ET decrease of 9–16% with the differences caused variation in temperature among the growing seasons (Bernacchi et al., 2007).

Water deficit conditions are likely to occur under increasing variation of precipitation and will increase the importance of understanding the interactions of CO_2_ enrichment with climatic factors of water supply and evaporative demand. An advantage of elevated CO_2_ will be evident first on reduced *g*_s_ which in turn leads to enhanced soil water conservation and less water-deficit stress detectable when crops are grown under conditions with periodic soil water deficit or under high evaporative demand. Reducing water-deficit stress has a positive impact on photosynthesis, growth, and yield and that has been documented for wheat (Wall et al., 2006) and sorghum (Ottman et al., 2001; Wall et al., 2001; Triggs et al., 2004). Under water deficit conditions, sorghum showed a positive biomass and grain yield response to the CO_2_ increases; however, the CO_2_ effect was not observed when the crop was grown under full irrigation (Ottman et al., 2001). The *g*_s_ of the sorghum plant was reduced by 32–37% (Wall et al., 2001) with a concurrent decrease in ET of 13% (Triggs et al., 2004).

### Impact of Ambient Air Temperature

Exposure to higher temperatures from both experimental evidence and simulation models shows the CO_2_–induced benefit to conductance diminishes as temperatures increase. Observations of leaf temperatures in controlled environment chambers with a twofold increase in CO_2_ showed soybean foliage temperatures increased 1–2°C, dry bean (*Phaseolus vulgaris* L.) by 1.5°C, and sorghum by 2°C (Pan, 1996; Prasad et al., 2002, 2006; Allen et al., 2003). Wand et al. (1999) conducted a meta-analysis on wild C_3_ and C_4_ grass species, grown with no stress, and observed that elevated CO_2_ reduced *g*_s_ by 39% in C_3_ and 29% in C_4_ species. The *g*_s_ to combinations of CO_2_, temperature, and VPD has been evaluated using crop simulation models (Allen, 1990). In these simulations, increasing CO_2_ from 330 to 800 μmol mol^-1^, increased foliage temperature by nearly 1°C with low air VPD, but showed an increase of 2.5–4°C with air VPD in the range of 1.5 and 3 kPa. Experimental observations on soybean showed canopies increased their conductance when exposed to progressively larger VPD (associated with higher temperature).

Increasing air temperatures will negate the positive effects of CO_2_ on plant growth, in soybean there was a 9% decrease in ET at 28/18°C but no reduction at 40/30°C (Allen et al., 2003), while in rice (*Oryza sativa* L.) there was a 15% reduction in ET at 26°C but an increase at 29.5°C (Horie et al., 2000). In general, increasing CO_2_ at moderate temperatures will create increased WUE; however, the positive effect diminishes as the temperature increases above the optimum temperature for the species.

There are offsetting effects between the increases in canopy temperature from the increased air temperature and the increased leaf area caused by the increase in CO_2_ resulting in very small changes in ET (Allen et al., 2003). An examination of Eq. 4 provides an assessment of the impact of a changing climate on crop ET. Kimball (2007) utilized data from Maricopa, Arizona to assess changes in temperature on alfalfa (*Medicago sativa* L.) ET and found only changing temperature, the reference ET increased 3.4% °C^-1^. When a constant relative humidity was evaluated, and temperature increased, annual ET increased 2.1% °C^-1^. With a change in absolute humidity, potentially caused by precipitation changes there was a decrease in ET by -0.2% per % change in absolute humidity. The feedbacks between transpiration and leaf temperature under changing CO_2_ have been evaluated by Boote et al. (1997) using a soybean growth model to show seasonal transpiration decreased 11–16% under irrigated conditions and 7% for rainfed environments, while ET decreased by 6–8% in the irrigated site and 4% for the rainfed conditions. The simulated WUE showed an increase between 53 and 61% and was attributed to prolonged use of soil water in the rainfed environments. These studies highlight the need to understand the interactions of soil water, CO_2_, and temperature during the growing season in or to develop more effective management strategies to cope with the changing climate.

### Solar Radiation

One component of the changing climate that is often overlooked, but extremely critical for growth is the solar radiation regime. A changing climate will increase clouds and potentially aerosols causing an increase in diffuse solar and PAR. Stanhill and Cohen (2001) found that over time there was a “solar dimming” in agricultural areas caused by the increasing presence to clouds and water vapor in the atmosphere. There have been reviews on this topic, (e.g., Ruimy et al., 1995; Kanniah et al., 2012, 2013) and revealed that changes in the solar radiation regime would affect photosynthesis and GPP. In a recent study, Gao et al. (2018b) evaluated the effect of clouds and aerosols on maize GPP and WUE and found that both RUE and WUE decreased linearly with increasing clearness, i.e., more direct PAR. Kromdijk et al. (2016) observed that the fluctuating light would actually increase productivity in plants because of the effects on photoprotection mechanisms while Slattery et al. (2018) found that C_4_ plants are most sensitive than C_3_ plants to fluctuating light conditions. They attributed this difference to changes in enzyme activities during light fluctuations. While in C_4_ plants, there was a greater sensitivity to variable light due to linkage between the bundle sheath C_3_ cycles and the mesophyll C_4_ cycles. Drewry et al. (2014) showed that changes in canopy architecture would have positive benefits on the overall productivity of crops and should be considered as a component in addressing future cropping systems. Changes in the radiation environment to more diffuse radiation increased both radiation-use efficiency (RUE) and WUE because of the more uniform light environment on the maize canopy. Changes in the solar radiation environment under climate change needs to be considered when evaluating the effects of temperature and precipitation.

## Cropping System Impacts on Water Use Efficiency

Cropping systems interact with climate with changes in phenology, growth, yield, and water use (Hatfield et al., 2011). The changes that are occurring and will occur under climate change will impact the efficiency of radiation capture (radiation use efficiency, RUE) and WUE. These two metrics are related through the dynamics of plant growth; however, if we examine WUE in response to climate variation, we must be aware that the efficiency of a plant canopy to intercept light will be affected by those same variables. These variables are not the only ones that affect WUE, earlier studies, e.g., alfalfa in response to ozone (O_3_) and water-deficit stress showed the interaction between these factors (Temple and Benoit, 1988). Increasing O_3_ reduced WUE because of the effect on leaf senescence and maintenance of leaf area of the alfalfa canopy. This illustrates that climate change impacts could arise from many different parameters that would affect WUE.

Over the recent years, studies on the impact of climate change and WUE have made use of historical observations of the growing season conditions coupled with physiological responses of different crops to temperature and CO_2_ (Guoju et al., 2013a,b). On potato (*S. tuberosum* L.), Guoju et al. (2013b) used controlled experiments to manipulate temperatures to define the relationship between temperature and WUE. They found WUE increased as temperatures increased to 1.5°C above normal and then began to decrease. Interestingly, they found that WUE began to decrease linearly with increases in annual precipitation above 310 mm. They proposed that the combination of increased temperatures and precipitation affected the respiration rate of potato which directly influenced the productivity of the plant. This concept was proposed by Reichstein et al. (2002) for evergreen trees demonstrates that agriculturalists focus on the climate effects on respiration as a factor contributing to WUE. In a similar study conducted in the semi-arid of northwest China, Guoju et al. (2013a) found for wheat, potato, and maize there was an increase of 1.6°C in the period from 1990 to 2009 compared to 1960–1969 and a decrease in annual precipitation of 105.6 mm. They showed that WUE increased in the 1990–2009 period in wheat by 0.0011 mm m^-2^ yr^-1^, potatoes by 0.00045 mm m^-2^ yr^-1^, and maize by 0.0012 mm m^-2^ yr^-1^ compared to the previous values. This response could be expected, if the temperatures during the growing season were not above the optimum for the specific crop.

## Cultural Practices at the Canopy Scale

### Fertilizer Application and Mulching

Climate change can extend beyond the direct impacts on photosynthesis and water use by canopies to the indirect impacts related to changes in cultural practices that would affect how crop canopies respond to climate variation. The framework for these changes can be shown in Figure 4, when we separate the soil and plant components in water use. For example, Li et al. (2018) evaluated plastic and straw mulch on WUE of potato and found plastic mulch increased productivity by 24% and straw mulch by 16%. This resulted in an increase of WUE of potato by 29% under a plastic mulch and 6% under the straw mulch. The effectiveness of the mulching practices on WUE were increased when precipitation was less than 400 mm and decreased when precipitation was above 400 mm. They found WUE of potato was affected by seasonal air temperature, precipitation, baseline soil fertility, and fertilizer management. Seasonal temperatures between 15 and 20°C showed the highest WUE but declined when temperatures were above or below this range (Li et al., 2018). In a study that combined plastic mulch with plant density on maize, Liu et al. (2014) found that different mulches did not affect WUE, but plastic mulch increased WUE compared to no mulch and this additional water saved because of the reduced soil water evaporation was able to support a higher plant population. The effects of mulch on WUE was reviewed by Zhang et al. (2017) and overall, mulch increased WUE by 61% because of the change in the water balance and the increased productivity of the maize crop.

Adding crop residue to the soil surface has shown benefits in decreasing soil water evaporation and increasing WUE in semi-arid regions. Ali et al. (2018) evaluated different soil management practices and found adding wheat residue at 5 t ha^-1^ coupled with an irrigation of 350 mm increased soil water availability compared to no residue and increased grain yield by 62% and WUE by 35%. They found that the presence of the wheat residue increased rainfall-use efficiency by 50% because of the reduced soil water evaporation. Wang et al. (2014) evaluated planting pattern and irrigation on wheat in the North China Plain and found a combination of furrow ridge planting combined with 135 mm of irrigation increased WUE by nearly 14% and suggested that this strategy would provide a more efficient production system in water-limited environments. Ibrahim et al. (2015) showed that mulching and micro-dosing of NPK fertilizer increases WUE in low-input agriculture in a semi-arid climate.

In tall fescue (*Festuca arundinacea* Schreb.), Kunrath et al. (2018) found that limiting nitrogen to the crop had a negative effect on WUE because the transpiration decreased relative to soil water evaporation. Similar results for alfalfa when plant stands were decreased, WUE decreased because of the increased soil water evaporation component (Kunrath et al., 2018). They suggested that understanding the interactions between nitrogen status and water deficits would be necessary to improve WUE.

### Irrigation

Another manipulation of the microclimate of the crop is to apply irrigation as a water supply to overcome water deficits. The impact on WUE could be substantial, if, the amount of water applied greatly improved the production compared to the amount of water use by the crop. Fan et al. (2018) conducted a meta-analysis on 49 experiments of irrigated wheat and cotton throughout China under furrow and micro-irrigation systems to determine the optimum water use level to achieve maximum WUE. If the goal is to maximize WUE rather than yield, across these studies, water use for wheat could be reduced by 30% with a grain yield decrease of only 15%; however, in cotton water use of 51% was linked with a yield reduction of 52%. The adoption of micro-irrigation was able to reduce wheat water use by 23% and increase yield by 37%, while in cotton this practice reduced water use by 37% and decreased yield by 21%. Adoption of micro-irrigation system reduces the soil water evaporation from between the plant rows early in the season and limits almost all the evaporation component from the canopy. These changes have a positive effect on WUE in areas with irrigated crops and demonstrates that WUE can be changed by water management of the system.

### Crop Arrangement

Manipulation of row spacing will affect the partitioning of the soil water evaporation and the transpiration of the canopy. Narrow rows would reduce the time the soil is not covered (Figure 4) and in theory would increase WUE. Barbieri et al. (2012) compared 35 and 70 cm rows of maize and found there was no difference in seasonal totals of ET because differences between row spacing diminished as the maize plant developed; however, the narrow row spacing increased WUE by 17% with significant effects when the crop was nitrogen and water limited but had no effect if the crop was irrigated and well-fertilized (Barbieri et al., 2012). As a climate adaptation strategy, reducing row spacing would increase WUE in water limited environments or under rainfed environments with increasing variability of rainfall during the growing season. Gregory et al. (2000) showed using simulation models, reduction of row width was an effective strategy in rainfed production regions of the world because of the effect on soil water evaporation. They showed that changing row spacing was most effective in areas with clay soils with frequent rain events and low atmospheric demand and would least effective in sandy soils with variable rain events and high evaporative demand.

### Crop Rotations and Mixed Crop Stands

To cope with climate change, one adaptive strategy would be to diversify the crop rotation to increase the resilience of the overall cropping system. Álvaro-Fuentes et al. (2009) compared four rotations in northeastern Spain to determine if adding a more diverse rotation of wheat and barley (*Hordeum vulgare* L.) would increase WUE. They used four rotations over a 6-year period, a wheat monoculture, a barley monoculture, wheat-barley-rapeseed (*Brassica napus* L.), and wheat-barley-vetch (*Vicia sativa* L.). Rainfall variation among growing seasons was the main determinate of water use and WUE. There was a failure of the rapeseed and vetch to produce a crop in several of the years, that impacted the WUE of the overall rotation system compared to the monoculture systems (Álvaro-Fuentes et al., 2009). Franco et al. (2018) examined an intercropping system consisting of peanut, watermelon [*Citrullus lanatus* (Thunb.) Matsum. & Nakai], okra [*Abelmoschus esculentus* (L.) Moench], cowpea, and pepper (*Capsicum annuum* L.) planted alone or in various intercropping combinations. In a low fertilizer input system in Texas. Peanut showed an increased WUE from 0.00015 kg plant^-1^mm^-1^ when grown in monoculture to 0.00022 kg plant^-1^mm^-1^ when grown in an intercropping system, watermelon and okra both showed similar positive responses to intercropping. They suggested that intercropping system would offer advantages for more efficient water use in water-limited environments.

### Agroforestry

Another strategy is to utilize agroforestry systems (AFS), where woody perennials (i.e., trees or shrubs) or perennial forbs are grown with annual crops on the same parcel of land and during same or different time periods. AFS systems comprise silvopastures, alley cropping, riparian buffers, windbreaks, parklands, home gardens, and fallow systems, among many others (Sauer and Hernandez-Ramirez, 2011; Nair et al., 2017). One theory is that the AFS annuals and perennials utilize different resource pools, i.e., there is a spatial complementarity. The roots of the tree component reach deeper soil layers than the annual crops, thus exploiting unused water and nutrient resources. At the same time the upper tree canopy provides shade to crops, which reduces evaporative water loss and water stress by generating a favorable micro-climate. The opposite would be the spatial competition, where the different AFS components compete for the same resources. Tree roots exploit the same soil layers than annual crops, the tree canopy reduces incoming PAR, and negatively affects rainfall distribution (Monteith et al., 1991; Ong et al., 1991). Both, spatial complementarity and competition, can occur and may change over time. For example, trees may cause a decrease of the groundwater table, which may lead to tree dieback under extreme climatic conditions (Song et al., 2015). Adverse effects can be partly ameliorated with additional management efforts such as tree root barriers, tree canopy pruning, use of different tree species, tree density and spatial arrangement, and effective use of run-off water (e.g., Ong et al., 1991; Droppelmann et al., 2000; Muthuri et al., 2009). These interactions eventually alter WUE. However, studies on WUE in AFS are scarce to fully understand all interactions and implications to WUE in the different types of AFS. Droppelmann et al. (2000) showed higher and lower WUE values among two seasons in sorghum and cowpea planted in an *Acacia saligna* (Labill.) H. Wendl. alley cropping system in semi-arid Kenya compared to monocrop annuals. Eastham et al. (1990) found that WUE in silvopastures of both, forage and trees, changed with tree density, but the authors did not compare WUE with single pasture systems. Muthuri et al. (2009) found higher chlorophyll content in monocrop maize than in AFS maize in Kenya, and that WUE and chlorophyll content are correlated. Perhaps a better way to look at WUE in AFS is the WER (see above). Bai et al. (2016) found in a semi-arid climate in northeast China consistently higher WUE in monocropping systems of sweet potato [*Ipomoea batatas* (L.) Lam.], peanut, and millet [*Setaria italica* (L.) P. Beauvois] compared to intercropped with apricot trees. However, overall WER was greater than 1 owing to the similar WUE of apricot in AFS and monocrop settings, indicating that overall yield to water use ratio was improved in AFS. Despite the need to further investigate WUE in AFS, there are promising results indicating that under good management practices AFS may ameliorate water use in water-limited environments, such as in sub-humid – arid climates, or during the dry season in the humid tropics.

## Water Use Efficiency Trends

Basso and Ritchie (2018) suggested that WUE has increased over time because the grain yields have increased while water use has remained relatively constant. Nagore et al. (2017) compared an older maize hybrid with more recently released hybrids and found the more recent hybrids had a WUE of 25.1 kg ha^-1^ mm^-1^ compared to 23.1 kg ha^-1^ mm^-1^ for the older hybrid. The more recent hybrids also showed a greater advantage in WUE all at soil water contents over the course of this study. The plant parameter that showed the advantage to increasing WUE was kernels per plant. The resilience of genetic material to stress, e.g., temperature or water, will provide the newer genetic material with greater WUE.

Increasing WUE under climate change will result from two fronts. First, being able to identify genotypes that have high assimilation rates under temperature and water-deficit stress. There are a range of techniques that can be used at both the leaf and canopy level and development of tools oriented toward phenotypic screening relative to WUE would pay dividends in terms of advancing our knowledge. We still face the challenge of being able to quantify the differences among plants in their response to temperatures above the optimum, water deficits, and increasing CO_2_ and more importantly the interactions among these three factors. Second, we need to realize that there are a range of management practices we can adopt that will reduce soil water evaporation and shift the water use by the crop to more transpiration to limit the exposure of the plant to water-deficit stress and maintain productivity at the highest level possible. We can cope with climate change by understanding the physical and biological factors that interact to create a high WUE.

## Author Contributions

All authors listed have made a substantial, direct and intellectual contribution to the work, and approved it for publication.

## Conflict of Interest Statement

The authors declare that the research was conducted in the absence of any commercial or financial relationships that could be construed as a potential conflict of interest.
